# Physical, Psychological and Social Secondary Consequences of the COVID‐19 Pandemic in Turkish University Students

**DOI:** 10.1002/gch2.202100098

**Published:** 2022-05-11

**Authors:** Cemil Örgev, Gülşah Kınalı

**Affiliations:** ^1^ Department of Health Management Sakarya University of Applied Sciences Yeni Mah., Alaağaç Jandarma Karşısı, Akyazı Sakarya 54400 Turkey; ^2^ Department of Physiotherapy and Rehabilitation Sakarya University of Applied Sciences Yeni Mah., Alaağaç Jandarma Karşısı, Akyazı Sakarya 54400 Turkey

**Keywords:** addiction, anxiety, COVID‐19 pandemic, musculoskeletal pain, physical inactivity, socio‐economic effects, University students

## Abstract

This study aims to investigate the effect of the COVID‐19 pandemic on university students and their families. A total of 274 students participated in this study. A questionnaire is developed regarding the physical, psychological, and social effects of the COVID‐19 pandemic, and sent to the students via e‐mail. According to the results of the study, students who contracted COVID‐19 experienced significantly higher musculoskeletal pain (MSP) (*p* = 0.01). Students with increased propensity for harmful habits experienced significantly more MSP (*p* = 0.01). Anxiety levels of students who experienced MSP were significantly higher (*p* = 0.01). Students living in an inadequate home environment were significantly more prone to harmful habits (*p* = 0.01). The anxiety level of the students who experienced domestic unrest was high (*p* = 0.02). The home environment of students with economic difficulties was significantly inadequate (*p* = 0.01). The anxiety levels of the students whose participation in sports and art activities decreased, were high (*p* = 0.04). The home environment of the students who reported that they were positively affected by distance education was significantly inadequate (*p* = 0.03). The authors suggest that physiotherapy, nutrition and psychological assistance services should be provided to students, scholarship opportunities and extracurricular activities (art and sports activities) should be increased.

## Graphical abstract

University students constitute a vulnerable segment of society that is greatly affected by sudden changes. In this study, the secondary effects of the Covid‐19 pandemic on university students are evaluated and the necessary solutions are listed. The authors hope that this study will inform management and interventions that should be applied in similar unexpected situations in future.

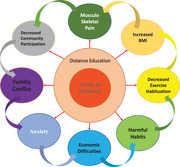

## 1 Introduction

There have been epidemics and disasters throughout human history, triggering long‐term physical, psychological, and social problems as well as primary effects such as death and injury. Currently, with the COVID‐19 pandemic, there is a need for information about its secondary effects. University students are a vulnerable segment of society to sudden changes. Therefore, we designed our study to evaluate the secondary effects of the COVID‐19 pandemic on university students and their families.

Humanity was not prepared for the COVID‐19 pandemic. Many important difficulties in such areas as controlling the pandemic, taking protective measures, and providing medical services have been overcome. COVID‐19 is characterized by its effects on multiple tissues and organs throughout the body, especially lung damage. In addition to pulmonary fibrosis, some patients may have long‐term dysfunction in the heart, liver, kidney, nervous, and immune systems.^[^ ^1^ ^]^

### 1.1 Distance Education and Home Environment in the COVID‐19 Pandemic

The COVID‐19 pandemic has sent the education industry to an unexpected location, the home. It would be wrong to describe the change in the education process as simply a switch to online education. Both online and distance education were started at many universities, and students had to study from home due to quarantine conditions. Scientific studies have drawn attention to the following challenges regarding distance education during COVID‐19: unstable internet connections, insufficient learning resources, power outages, ambiguous learning contents, overloaded lesson activities, limited teacher support, poor peer communication, conflicts with household responsibilities, poor learning environments, financial issues, physical health problems, and mental health struggles.^[^ ^2^ ^]^ In addition, there have been difficulties for educators in this process, as they have had to exert intense physical and emotional efforts to maintain contact with the student.^[^ ^3^ ^]^ In addition, distance education has not been “accessible to everyone.” In this case, it can be said that the COVID‐19 pandemic period causes situations where equal opportunities for education cannot be achieved.^[^ ^4^ ^]^

### 1.2 Digital Addiction, Physical Inactivity, and Obesity

In order to reduce the spread of the COVID‐19 pandemic and prevent people from being exposed to the virus, various authorities have recommended that the public stay at home. Restriction of outdoor activities has caused changes in individuals’ routine daily activities, including regular physical activity and exercise. Staying at home for a long time can lead people to spend excessive amounts of time on inactive behaviors such as playing digital games, watching television, and using mobile devices.^[^ ^5^ ^]^ In this respect, we can say that social isolation changes our lifestyle, causing us to stay at home more, increasing our physical inactivity, and devoting more time to technology. Studies of university students during the pandemic have shown that university students’ addiction to digital games has increased.^[^ ^6^ ^]^ It has also been reported that problematic Internet use also increased during the COVID‐19 pandemic period, and that studies should be carried out to protect adolescents and adults from the risk of such use.^[^ ^7^ ^]^ In previous studies, it was determined that students with high depression levels were more addicted to social media.^[^ ^8^ ^]^ Studies conducted during COVID‐19 have also shown that university students with high depression levels tend to use more social media.

When the relationship between COVID‐19 and obesity is examined, two types of connections emerge. Obesity is a risk factor for mortality due to COVID‐19. On the other hand, it may also be a result of changing lifestyles due to social isolation from the pandemic. The presence of chronic diseases, such as underlying obesity, worsens the patient's condition. Among infected patients, the elderly and those with multiple comorbidities have shown associations with severe disease processes. Staying at home as a result of increased distance from social environments has reduced people's physical activity levels, increased their energy intake, and prompted behaviors such as stocking food to minimize going out of the house for shopping.^[^ ^9^ ^]^

### 1.3 Musculoskeletal Problems

Acute viral diseases often present with myalgia and fatigue, and these symptoms were also seen with influenza during the 1918 and 2009 H1N1 pandemics and during the SARS epidemic.^[^ ^10^ ^]^ In these types of diseases, the focus is on the immediate response to acute illness, but subsequent consequences are ignored. In a small study of 22 subjects (21 healthcare workers) infected during the SARS epidemic, chronic post‐SARS syndrome consisting of fatigue, widespread myalgia, depression, and non‐restorative sleep was reported to last for almost 2 years.^[^ ^11^ ^]^

### 1.4 Economic Difficulties

The phrase “COVID‐19 does not discriminate” has been repeated on several occasions. However, this is a dangerous myth that ignored the growing vulnerability of the most socially and economically deprived. In response to the pandemic, policy makers had to implement quarantine practices, an action unmatched since World War II. In summary, a combination of factor makes the most economically disadvantaged particularly vulnerable to COVID‐19. Possible causal mechanisms include greater exposure to the virus, poverty‐related stress and comorbidities, and reduced access to healthcare. To address the vulnerabilities of the most economically disadvantaged segments of society, policymakers should introduce long‐term legislation to improve social welfare.^[^ ^12^ ^]^

The COVID‐19 pandemic has caused a major economic shock worldwide due to job cuts and closures resulting from social distancing measures. COVID‐19 has caused serious and acute losses in many economies around the world due to the disease and social distancing regulations mandated by governments. It is difficult to predict the impact and duration of the economic crisis resulting from the pandemic on individual households, because the duration of the crisis, that is, the length of stay‐at‐home orders, as well as the affected sectors and aftermath, entail many uncertainties.^[^ ^13^ ^]^ This situation is similar across much of the world. Important moments like these require a strong and robust governance system in the fields of health, business, government, and society at large, and immediate support measures should be initiated and adapted for those whose circumstances may be worsened by the situation. Medium‐ and long‐term strategies are needed to stabilize and motivate the economy during this recession.^[^ ^14^ ^]^

While the psychological and social impacts of the COVID‐19 pandemic share some similarities with those of past disasters, there are also major differences in support of response and recovery for both individuals and communities. COVID‐19 has painfully exposed the existing and ongoing health inequalities in societies and has had the most severe impact on the lives of people living in deprivation or facing difficult socio‐economic conditions. These individuals may be more likely to develop chronic illnesses and mental illnesses.^[^ ^15^ ^]^

### 1.5 Family Relations

Individuals and families have not only deal with the threats to their own health from COVID‐19 by trying to avoid infection and survive, but have also suffered serious losses. For many, there is the loss of family members (which is many cases occurs in circumstances preventing family contact, which is unusual in this age). Almost everyone has worries and other feelings about potential losses. Combined with other problems accompanying the pandemic (for example, rising unemployment and financial fragility), loss and potential loss are ubiquitous. It is clear that there are additional risks in couples and families who are at risk of violence, conflict, or other relationship difficulties. Not surprisingly, early data from China point to an increase in divorce rates during times of quarantine. Additional difficulties may arise for families dealing with troubled family members with the help of others who are not currently present. In other families, seemingly successful family transition processes, such as young adults leaving home to establish their own identities, have suddenly been radically reversed, giving rise to countless problematic possibilities. There is also a challenge now of interruptions in daily life for those who depend on rituals for connection, whether church, alcoholics anonymous meetings, or family dinners. Research shows that maintaining such regular and reliable rituals can be central in distinguishing those who are injured from those who remain resilient in difficult times. As is often the case with dire events, its effects are more pronounced for those with the least financial resources.^[^ ^16^ ^]^

In addition, studies examining the impact of crisis situations on crime and violence rates have revealed that reports of domestic violence to police departments increase significantly after events such as natural disasters.^[^ ^17^ ^]^

### 1.6 COVID‐19 and Anxiety

Recent studies of the psychological and social effects of COVID‐19 have also shown that this disease causes radical changes in the vital conditions of many societies and is associated with negative psychological outcomes. For example, in a study conducted with 1210 participants during the COVID‐19 outbreak in China, 16.5% of the participants had moderate to severe depression symptoms, while 28.8% had moderate to severe anxiety symptoms. The same study reported that women were affected more negatively by the psychological outcomes of the epidemic than men; their scores on measures of stress, anxiety, and depression were significantly higher. This may be due to increased workload. In addition, groups with lower education showed higher depression symptoms; it was found that other demographic variables such as age, parenting status, and marital status were not associated with anxiety and depression levels. Another striking finding of related research is that individuals who stated that they could better access reliable messages and protective measures (hand hygiene and mask) of local governments were found to have significantly lower levels of anxiety and depression during the social isolation process. However, studies have found a connection between anxiety and hopelessness during the COVID‐19 period.^[^ ^18^ ^]^ There have, however, been limited studies of the COVID‐19 period and anxiety levels among university students. In a study conducted in Turkey using the COVID‐19 Phobia Scale, high levels of coronaphobia were found in medical faculty Term 5 and Term 6 students, both on the sub‐dimensions and overall.^[^ ^19^ ^]^

### 1.7 Harmful Habits

Alcohol use has increased during the past pandemic period. It is inevitable that trying to keep up with the changing daily routines with the epidemic affects spiritual life. In addition, people experiencing the death of a family member due to COVID‐19 may not have the opportunity to conduct a burial according to their cultural traditions, which can disrupt the grieving process, posing a risk for consequences of the failed grieving process. Considering all these, the COVID‐19 pandemic is a strong risk factor for mental and behavioral disorders such as depression, post‐traumatic stress disorder, and alcohol use disorder. In the severe acute respiratory syndrome (SARS) epidemic in Asia in 2003, it was found that among the hospital staff working in departments affected by SARS, participants who were in quarantine or who lost one of their relatives due to SARS exhibited symptoms of post‐traumatic stress disorder 2–3 times higher than normal.^[^ ^20^ ^]^ Arpacıoğlu et al., examining changes in alcohol use during the coronavirus epidemic, reported that alcohol and cigarette use decreased during the epidemic.^[^ ^21^ ^]^ College students are more vulnerable to stress and depression, constituting a vulnerable group because of their mental health and tendency to engage in unhealthy behaviors. At the same time, risky behavior and excessive alcohol consumption are no exception in this age group. Additionally, evidence suggests that university students are at a higher risk of problem alcohol consumption than non‐students in the same age group. As a result, efforts to reduce stress will be reflected in the reduction of depressive disorders and excessive alcohol consumption among students. Alcohol consumption is closely related to the mental health of university students.^[^ ^22^ ^]^

### 1.8 İnterventions in Turkey during COVID‐19

COVID‐19 cases were first seen in Wuhan, in Hubei Province of China. The first case in Turkey was announced by the Ministry of Health of the Republic of Turkey on March 10, 2020.^[^ ^23^ ^]^ On March 16, it was announced that face‐to‐face education was suspended, and the distance education process started on March 23. In this period, teachers’ interactions with students, communication, and methods of realizing the lessons have changed.^[^ ^24^ ^]^ During the pandemic in Turkey, the transition to distance education in universities has been gradual. Of 189 universities, 121 (64%) switched to distance education on March 23, 2020, 41 (21.6%) on March 30, 2020, and 25 (13.2%) on April 6, 2020. While the number of courses to be formally opened in higher education institutions in the 2019–2020 spring semester is 736341, with the transition to distance education, 663808 courses were opened and 90.1% of the courses were given via distance education. By area, such courses made up 91% of those in the social sciences, 78% in engineering sciences, 77% in natural sciences, and 54% in the health sciences. Diversity has also been provided in student assessment methods in the distance education process in higher education institutions. Of the assessments, 66% chose online exams, 91% homework, 83% projects, and 58% quizzes; more than one assessment method is used in most courses.^[^ ^25^ ^]^

During the COVID‐19 epidemic, Turkey conducted studies to eliminate and prevent the primary and secondary effects of the epidemic. Social support groups were formed to prevent individuals from feeling lonely, psychosocial counseling lines were set up to support individuals with psychological problems, mental health support systems, immigration, and moral support centers were established to provide services to individuals who have immigrated as well as adequate specialists, a rapid response system was created to address the problems of children with special needs, and a support center for children with special needs was established. In order to prevent disadvantaged individuals from experiencing difficulties due to not being able to reach services during the epidemic, an orbit reader was distributed for visually impaired students, and a literacy learning kit was published for hearing‐impaired students. Museums and libraries have been made accessible to everyone electronically to prevent the negative effects of social isolation.^[^ ^26^ ^]^

### 1.9 Research Questions

Although we encountered COVID‐19 at an unexpected time, after the known acute effects of COVID‐19, according to previous studies and official and informal data from the field, COVID‐19 may have long‐term secondary effects.^[^ ^1^, ^2^, ^3^, ^4^, ^5^, ^6^, ^7^, ^8^, ^9^, ^10^, ^11^, ^12^, ^13^, ^14^, ^15^, ^16^, ^17^, ^18^, ^19^, ^20^, ^21^, ^22^ ^]^ The research question of this study was whether the secondary effects of COVID‐19 identified in previous epidemics are also common among university students and whether these effects are related to one another.

## 2 Experimental Section

### 2.1 Questionnaire Design

The COVID‐19 process was ongoing at the time of the study. There has not yet been a standardized survey investigating the secondary effects of COVID‐19 among university students. Therefore, the questionnaire for this study was prepared based on previously published studies^[^ ^1^, ^2^, ^3^, ^4^, ^5^, ^6^, ^7^, ^8^, ^9^, ^10^, ^11^, ^12^, ^13^, ^14^, ^15^, ^16^, ^17^, ^18^, ^19^, ^20^, ^21^, ^22^ ^]^ on the secondary effects of epidemics and the physical, psychological, social, and problems of university students during the COVID‐19 era, as well as input from the research team, statisticians, clinicians, and the target population. The questionnaire consisted of 12 parts and 35 sub‐questions in total. Questionnaire sections were divided as follows: demographic information, body mass index change, musculoskeletal pain, exercise habits, information on covid‐19 disease and losses, relations in this process, technical sufficiency of the house for distance education, harmful habits, economic situation, thoughts on distance education and access to services. The research questionnaire was administered to five young volunteers from the target population. In this pilot test, participants were asked to provide feedback on the understandability of the questionnaire, ease of implementation, whether the interface transitions worked, and the time required to complete the questionnaire. Participants stated that the measurement was easy to use and understand, and the completion time was reasonable. The questions were prepared in Turkish and English was also available for foreign students in need. No request was received to answer the research questionnaire in English.

### 2.2 Study Participants

There were 22 882 students at the university where the study was conducted. The online questionnaire was sent to all students by the university's communication coordinatorship. Ethics committee approval was obtained from Sakarya University of Applied Sciences (No. 100/8764) before starting the study. A total of 274 volunteer students participated in this study. Of the students participating in the study, 50% were female and 50% male (**Table** **1**). The average age of the students was 21.93 ± 4.52 years. It was determined that the average height of the students was 170.98 ± 9.91 cm and the average weight was 69.33 ± 17.63 kg. It was calculated that the BMI levels of the students were 23.51 ± 4.70 (**Table** **2**).

**Table 1:** Gender distribution of students

		*n*	%
Gender	Male	137	50.00%
	Female	137	50.00%

**Table 2:** Physical characteristics of students

Measurement	*N*	Mean	S.D.	Minimum	Maximum
Age	274	21.93	± 4.52	18.00	54.00
Height	274	170.98	±9.91	150.00	200.00
Weight	274	69.33	±17.63	40.00	145.00
BMI	274	23.51	±4.70	15.24	43.30

### 2.3 Statistical Analysis

Descriptive statistics are presented as frequencies, percentages, means, and standard deviations. In this study, the Kruskal‐Wallis test was used to examine the differences in the measurements of the groups. The chi‐square test was used to compare the measurements in the groups as percentages. In the study, *p*‐values less than 0.05 were considered statistically significant (α = 0.05). Analyses were performed using SPSS 25.0.

## 3 Results

During the pandemic process, 56.6% of the students stated that their BMI levels increased. 42% of the students reported that they experienced musculoskeletal pain that they had not felt before. 23.7% of students reported that their exercise habits increased. 71.9% of the students reported that their home environment was sufficient for distance education during the pandemic period. 34.3% of the students reported that their tendency to harmful habits increased during the pandemic process. 61.3% reported having financial difficulties. 1.1% of students reported that they were positively affected by distance education. 90.5% of the students reported that the level of anxiety increased during the pandemic process, and 1.8% reported that they received expert support due to anxiety. 22.3% of students reported that they caught COVID‐19. While the death rate due to COVID‐19 in the families of the students was 1.1%, the death rate from the close relatives or friends of the students was 26.6% (**Table** **3**).

**Table 3:** Distribution of answers to questions

Questions	Answers	n	%
Has there been a significant change in your body weight during the pandemic?	Increased	155	56.6%
	Decreased	46	16.8%
	No change	73	26.6%
Have you experienced musculoskeletal pain that you have not felt before during the pandemic process?	Yes	115	42.0%
	No	159	58.0%
Has your exercise frequency changed during the pandemic?	Increased	65	23.7%
	Decreased	121	44.2%
	No change	88	32.1%
Have you caught Covid‐19?	Yes	61	22.3%
	No	213	77.7%
Has there been a death in your family due to Covid‐19?	Yes	3	1.1%
	No	271	98.9%
Have any of your close relatives and friends died due to Covid‐19?	Yes	73	26.6%
	No	201	73.4%
Was your home environment sufficient for distance education during the pandemic process?	Yes	197	71.9%
	No	77	28.1%
Has there been a harmful habit that your tendency has increased during the pandemic process?	Yes	94	34.3%
	No	180	65.7%
Did you and your family experience economic difficulties during the pandemic process?	Yes	168	61.3%
	No	106	38.7%
How has distance education affected you?	Positive	3	1.1%
	Negative	195	71.2%
	No change	76	27.7%
Has there been domestic unrest in the pandemic?	Yes	95	34.7%
	No	179	65.3%
Has your anxiety level increased during the pandemic process?	Yes	248	90.5%
	No	26	9.5%
If your anxiety level has increased, have you received expert support?	Yes	5	1.8%
	No	269	98.2%

Changes in the exercise habits of the participants were not significantly related to their BMI levels (**Table** **4**). The BMI levels of the students whose exercise habits increased, decreased, and did not change (*p* = 0,28, *p* > 0,05).) are shown in Table 4.

**Table 4:** The relationship between the changes in the exercise habits of the participants and the changes in their body mass index levels

Has your exercise frequency changed during the pandemic?	*n*	x�	S.D.	*p*
Increased	65	23.16	4.6	0.28^a)^
Decreased	121	24.02	5.05	
Has not changed	88	23.06	4.21	
Total	274	23.51	4.7	

^a)^ Kruskal‐Wallis test

The students’ status of contracting COVID‐19 was significantly related to their experience of musculoskeletal disease pain: The rate of catching COVID‐19 was higher in students who stated that they had muscle system disease pain (*p* = 0.01). Moreover, an increase in the harmful habits of students is significantly related to the pain of muscle system diseases: The rate of increase in harmful habits was higher in students who stated that the reason for the difference was pain due to muscular system diseases (*p* = 0.01). There is also a significant relationship between the students’ experience of musculoskeletal system pain during the pandemic and their anxiety levels, such that individuals experiencing musculoskeletal pain had higher levels of anxiety (*p* = 0.01). It was observed that the rate of increase in students’ harmful habits was significantly related to an adequate home environment. It was determined that the rate of increase in harmful habits was higher in students who stated that the reason for the difference was that the home environment was not suitable (*p* = 0.01). There is also a significant relationship among the participants between the level of anxiety and the experience conflict and violence in family relations during the pandemic. It was observed that the anxiety levels of individuals who experienced conflict and violence in family relations during the pandemic process were higher (*p* = 0.02), and that there is a significant relationship between economic difficulty and home environment adequacy, such that the home environments of the participants who had economic difficulties were more inadequate (*p* = 0.01; **Table** **5**).

**Table 5:** a) The relationship between having covid‐19 and having MSP. b) The relationship between harmful habits and MSP. c) The relationship between MSP and anxiety. d) The relationship between family confilict and anxiety. e) The relationship between harmful habits and home environment sufficiency. f) The relationship between economic hardship and home environment sufficiency

a) Have you caught Covid‐19?		Have you experienced a pain in the musculoskeletal system that has not been felt before during the pandemic process?		*p*
		Yes	No	
Yes	*n*	43	18	0.01^a)^
	%	70.50%	29.50%	
No	*n*	72	141	
	%	33.80%	66.20%	

b) Has your tendency to harmful habits increased during the pandemic process?		Have you experienced a pain in the musculoskeletal system that has not been felt before during the pandemic process?		*p*
		Yes	No	
Yes	*n*	57	37	0.01^a)^
	%	60.60%	39.40%	
No	*n*	58	122	
	%	32.20%	67.80%	

c) Have you experienced a pain in the musculoskeletal system that has not been felt before during the pandemic process?		Has your anxiety level increased during the pandemic process?		*p*
		Yes	No	
Yes	*n*	111	4	0.01^a)^
	%	96.50%	3.50%	
No	*n*	137	22	
	%	86.20%	13.80%	

d) Have you experienced family conflict during the pandemic process?		Has your anxiety level increased during the pandemic process?		*p*
		Yes	No	
Yes	*n*	91	4	0.02^a)^
	%	95.80%	4.20%	
No	*n*	157	22	
	%	87.70%	12.30%	

e) Has there been an increase in harmful habits during the pandemic process?		Was your home environment sufficient for distance education during the pandemic process?		*p*
		Yes	No	
Yes	*n*	54	40	0.01^a)^
	%	57.40%	42.60%	
No	*n*	143	37	
	%	79.40%	20.60%	

f) Did you experience economic difficulties during the pandemic process?		Was your home environment sufficient for distance education during the pandemic process?		*p*
		Yes	No	
Yes	*n*	106	62	0.01^a)^
	%	63.10%	36.90%	
No	*n*	91	15	
	%	85.80%	14.20%	

^a)^ Chi‐square test

A significant relationship was found between the sufficiency of the home environment for distance education and the satisfaction of the participants from distance education during the pandemic process. The home environment of the students who reported that they were positively affected by distance education was insufficient (*p* = 0.03; **Table** **6**). The anxiety levels of individuals whose participation in health and sports activities decreased during the pandemic process were higher than other participants. A significant correlation was found between participation in arts and sports activities and anxiety levels (*p* = 0.04; Table 6).

**Table 6:** a) The relationship between distance education and home environment. b) The relationship between participation in art and sports activities and anxiety

a) How has distance education affected you?	Was your home environment suitable for distance education during the pandemic process?			*p*
		Yes	No	
Positive	*n*	1	2	0.03^a)^
	%	33.30%	66.70%	
Negative	*n*	135	60	
	%	69.20%	30.80%	
No change	*n*	61	15	
	%	80.30%	19.70%	

b) How has your participation in art and sports activities changed during the pandemic process?	Has your anxiety level increased during the pandemic process?			*p*
		Yes	No	
Increased	*n*	13	2	0.04^a)^
	%	86.70%	13.30%	
Decreased	n	194	17	
	%	91.90%	8.10%	
No change	*n*	41	7	
	%	85.40%	14.60%	

^a)^ Chi‐square test

## 4 Discussion

The COVID‐19 pandemic has deeply affected university students, who represent a young and vulnerable segment of society. The aim of this study was to examine the secondary effects of the COVID‐19 pandemic on university students.

### 4.1 Family Communication Disorders

The COVID 19 pandemic period has been a process where people both try to protect themselves and face epidemic‐related deaths. However, there were also economic difficulties. Initial data from China showed that divorce rates increased during the pandemic. On the other hand, young adults could not leave the house when they would ordinarily do so due to university education, work, and so forth, and many rituals that support people spiritually cannot be performed.^[^ ^16^ ^]^

In addition, studies examining the impact of crisis situations on crime and violence rates revealed that reports of domestic violence to police departments increase significantly after events such as natural disasters. For example, during the COVID‐19 pandemic, in calls made to the police departments of Los Angeles to report a crime, there was a significant increase in domestic violence crime reports.^[^ ^17^, ^18^, ^19^, ^20^, ^21^, ^22^, ^23^, ^24^, ^25^, ^26^, ^27^, ^28^ ^]^ According to the results of our research, there was a significant relationship between family conflict and violence experienced by university students and their anxiety levels. Anxiety reports of students who reported experiencing domestic violence and conflict during the pandemic were higher (Table 3). According to the results of our study, domestic conflict and violence during the pandemic period increased students’ anxiety levels. During COVID‐19, high levels of corona phobia were detected in medical students, both in the sub‐dimensions and overall.^[^ ^19^ ^]^ In a study of Iranian medical students, the prevalence of anxiety was found to be 38% during the COVID‐19 pandemic.^[^ ^29^ ^]^ In a different study conducted with Chinese medical students, the rate of anxiety thought to be caused by the COVID‐19 pandemic in medical school students in China was found to be 24.9%.^[^ ^30^ ^]^ In our study, 90.5% of the students reported that their anxiety levels increased during the pandemic (Table 3). These results are consistent with those reported in the literature. According to our results of our study, The decrease in participation in sports and art activities increased the anxiety levels of the students (Table 6). In this case, it is necessary for universities to conduct studies for the development of measures to provide voluntary psychological support in order to maintain art and sports activities during pandemic and disaster periods, and to monitor the problems experienced by university students in the family and at home.

### 4.2 Economic Difficulties

Economic difficulties due to the COVID‐19 pandemic are significantly and positively correlated with distress, and experiencing more economic hardship is associated with higher levels of distress symptoms and a sense of danger.^[^ ^31^ ^]^ The COVID‐19 pandemic has further increased the vulnerability of the poor population. Working‐age adults and children are expected to be most affected by poverty, which has risen in the COVID‐19 crisis.^[^ ^31^, ^32^ ^]^ Researchers have reported that experiencing economic hardship during the COVID‐19 period increases the sense of distress and danger, and that people who are already poor will be more affected by this crisis. In our study, students who experienced economic difficulties also stated that their home environment was inadequate for participating in distance education (Table 5).

### 4.3 Students’ Thoughts on Distance Education and Home Environments

The COVID‐19 outbreak has led to the transition to both distance and online education. It would be wrong to describe the change in the education process by simply switching to online education. In this process, both online and distance education were started, and students had to study from home due to quarantine conditions. Researchers have drawn attention to the following challenges of distance education during the COVID‐19 era: unstable internet connections, insufficient learning resources, power outages, ambiguous learning contents, overloaded lesson activities, limited teacher support, poor peer communication, conflict with household responsibilities, poor learning environments, financial issues, physical health problems, and mental health struggles.^[^ ^2^ ^]^ In addition, there have been difficulties for educators, who have had to exert intense physical and emotional efforts to maintain contact with students.^[^ ^3^ ^]^ In addition, distance education has not been “accessible to everyone.” In this case, it can be said that the COVID‐19 pandemic period causes situations where equal opportunities for education cannot be achieved.^[^ ^4^ ^]^ In our study, we found that students with insufficient home environments were satisfied with their distance education (Table 5), perhaps because students who have economic difficulties and whose home environment is inadequate are satisfied with the flexible schedule provided by distance education because it affords them the opportunity to work.

### 4.4 Musculoskeletal Pains

Acute viral diseases often present with myalgia and fatigue. In these types of diseases, the focus is on the immediate response to acute illness, but subsequent consequences are ignored.^[^ ^10^, ^11^ ^]^ In our study, musculoskeletal pain was reported more widely in the students who had contracted COVID‐19. The anxiety levels and reported interest in harmful habits were also significantly higher in students with musculoskeletal pain (Table 5). In this case, we can say that the primary results experienced in the pandemic process have secondary consequences. The treatment of the disease during the pandemic and the fight against its secondary physical and psychological consequences should be considered to be interrelated processes.

### 4.5 Exercise Habituation and Body Mass Index (BMI) Level

Staying at home as a result of staying away from social environments has reduced people's physical activity levels.^[^ ^5^, ^9^ ^]^ Thus, we can say that social isolation changes our lifestyle, causes us to spend more time at home, experience greater physical inactivity, and devote more time to technology. Studies of university students during the pandemic have shown that university students’ addiction to digital games has increased.^[^ ^6^ ^]^ In previous studies, it was determined that students with high depression levels were more addicted to social media.^[^ ^8^ ^]^ Studies conducted during the COVID‐19 process have also shown that university students with high levels of depression tend to use more social media. According to our study, 56.6% of the students participating in the study stated that their BMI increased during the pandemic. Of the students, 44.2% stated that their exercise habits decreased (Table 3). The results of our study corroborate earlier results. Thus, education and support regarding nutrition and physical activity should be provided to students during and after the pandemic.

## 5 Conclusion

According to the results of our study, students experiencing economic difficulties during the pandemic process were negatively affected by distance education, their anxiety levels increased, their body mass index levels increased, and they experienced musculoskeletal system pain and decreased exercise habits. These secondary problems created by the pandemic have triggered another problem. According to the results of our study, it is necessary to combat the negative secondary effects of the COVID‐19 pandemic on university students. To this end, during the normalization process in universities, students should be given psychological support, scholarship opportunities can be increased for students experiencing economic difficulties, extracurricular activities (art and sports activities) should be increased, and preventive measures should be taken to prevent physical inactivity. In addition, physiotherapy and rehabilitation support should be provided at the university for musculoskeletal health.

## Conflict of Interest

The authors declare no conflict of interest.

## Data Availability

The data that support the findings of this study are available on request from the corresponding author. The data are not publicly available due to privacy or ethical restrictions.
